# Influenza A(H1N1)pdm09 Virus in Pigs, Réunion Island

**DOI:** 10.3201/eid1810.120398

**Published:** 2012-10

**Authors:** Eric Cardinale, Hervé Pascalis, Sarah Temmam, Séverine Hervé, Aure Saulnier, Magali Turpin, Nicolas Barbier, Johny Hoarau, Stéphane Quéguiner, Stéphane Gorin, Coralie Foray, Matthieu Roger, Vincent Porphyre, Paul André, Thierry Thomas, Xavier de Lamballerie, Koussay Dellagi, Gaëlle Simon

**Affiliations:** Le Centre de Recherche et de Veille sur les Maladies Emergentes dans l’Océan Indien, Sainte-Clotilde, Ile de la Réunion, France (E. Cardinale, H. Pascalis, S. Temmam, M. Turpin, J. Hoarau, C. Foray, M. Roger, K. Dellagi);; Centre de Coopération Internationale en Recherche Agronomique pour le Développement, Montpellier, France (E. Cardinale, J. Hoarau, C. Foray, M. Roger, V. Porphyre);; Institut de Recherche pour le Développement, Sainte-Clotilde (H. Pascalis, M. Turpin, K. Dellagi);; Centre National de la Recherche Scientifique–Université de Lyon, Lyon, France (S. Temmam);; Agence Nationale de Sécurité Sanitaire, de l’alimentation, de l’Environnement, et du Travail (ANSES), National Reference Laboratory for Swine Influenza, Ploufragan, France (S. Hervé, A. Saulnier, N. Barbier, S. Quéguiner, S. Gorin, G. Simon);; Clinique Vétérinaire, Saint-Louis, Ile de la Réunion (P. André);; Coopérative des Producteurs de Porcs de la Réunion, Saint-Pierre, Ile de la Réunion (T. Thomas);; and Université de la Méditerranée, Marseille, France (X. de Lamballerie)

**Keywords:** influenza A virus, pandemic, pigs, humans, zoonoses, H1N1 subtype, pandemic influenza, Réunion Island, viruses, H1N1, A(H1N1)pdm09

## Abstract

During 2009, pandemic influenza A(H1N1)pdm09 virus affected humans on Réunion Island. Since then, the virus has sustained circulation among local swine herds, raising concerns about the potential for genetic evolution of the virus and possible retransmission back to humans of variants with increased virulence. Continuous surveillance of A(H1N1)pdm09 infection in pigs is recommended.

Influenza A(H1N1)pdm09 virus, which caused the last influenza pandemic among humans, is a unique reassortant derived from swine influenza viruses of the triple reassortant swine North American lineage and the avian-like swine Eurasian lineage (*1*). Réunion Island, a tropical French overseas department in the southwestern Indian Ocean, was struck by the influenza pandemic during July–August 2009. The epidemic had a high attack rate in humans (estimated clinically at 12.5% and serologically at 40.0%) (*2*,*3*). A(H1N1)pdm09 virus was reported to cause a reverse zoonosis in pigs (*4*); thus, a long-term (2009–2011) serologic and virologic survey was designed to check for transmission of the virus to pigs on Réunion Island, where the pork industry is a key economic activity and no live pigs have been imported since 1978. At 6-month intervals, a local veterinary surveillance system conducts serologic surveillance for pathogenic swine influenza viruses (H1N1, H1N2, and H3N2) among local herds, and during the last 20 years, none have been detected.

## The Study

During a first step (November 2009–February 2010), seroprevalence rates for A(H1N1)pdm09 virus were assessed in 120 breeding pigs (>4 years old) from 57 farms. Blood was obtained from randomly selected pigs at the only slaughterhouse on the island, where pigs are held for <3 hours. We screened the samples for antibodies to influenza A viruses by using the ID Screen Antibody Influenza A kit (ID.vet, Montpellier, France), and titers were determined by using hemagglutination-inhibition (HI) assays (*5*) against all classical swine influenza viruses and A(H1N1)pdm09 virus (Table 1). Ninety-eight (81.7%; 95% CI 74.7%–88.5%) of the 120 serum samples were positive for A(H1N1)pdm09 virus (HI titers >20); the range of positive titers was 40–640, and 54.2% of the samples expressed high HI titers (160–640). Of the 98 serum samples, 5 reacted at low titer and with only 1 European A (H1N1) swine virus (titer <20), i.e., >4 dilutions lower than for A(H1N1)pdm09 virus, indicating cross reactivity (*6*). Thus, pigs from 47 (82.4%) of 57 tested farms had been infected by A(H1N1)pdm09 virus; the seroprevalence rate was 81%–100% for pigs on 79.0% of the farms. Farms with affected pigs were located throughout the island (Figure).

**Table 1:** Antigenic characterization of A(H1N1)pdm09-like influenza viruses isolated from pigs, Réunion Island, 2010*

Virus strain	HI titers† obtained with reference swine hyperimmune serum samples directed against				
	California/04/09‡	Sw/England/ 117316/86§	Sw/Cotes d'Armor/0388/09¶	Sw/Flandres/1/98#	Sw/Scotland/ 410440/94**
A/Sw/La Reunion/0164/10	1,280	160	40	<10	<10
A/Sw/La Reunion/110348/10	1,280	80	160	10	20
A/Sw/La Reunion/0167/10	640	80	40	<10	<10
A/Sw/La Reunion/110194/10	640	80	20	<10	10

*HI, hemagglutination inhibition. †HI tests were performed according to standard procedure (*5*).Titers are expressed as the reciprocal of the highest dilution of serum that inhibits 4 hemagglutination units of virus. ‡A(H1N1)pdm09 lineage. §Classical swine A(H1N1). ¶Avian-like swine A(H1N1). #Human-like reassortant swine A(H3N2). **Human-like reassortant swine A(H1N2).

**Figure:**
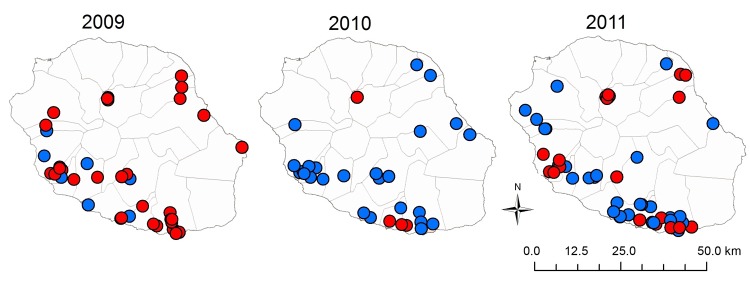
Location of farms tested for antibodies against influenza A(H1N1)pdm09 virus in serologic surveys, Réunion Island, 2009–2011. Blue dots, seronegative farms; red dots, seropositive farms.

In a second step (June 2010, when A(H1N1)pdm09 infection was no longer detected among humans), we tested whether the virus was still circulating among pigs born that year. To obtain nasal swab and blood samples for testing, we randomly selected 390 fattening pigs (25–27 weeks old) at the slaughterhouse; the pigs originated from 45 farms. At the time of sampling, the veterinary surveillance system did not report any clinical signs suggesting virus circulation among herds. However, ≈3.5% of the serum samples (9% of tested farms) contained antibodies to A(H1N1)pdm09 virus (HI titers 20–160). Nasal swab specimens from 6.7% (26/390) of pigs were positive for A(H1N1)pdm09 virus as determined by using a specific real-time reverse transcription PCR (rRT-PCR); the pigs originated from 13 (28.8%) farms (*7*). Two strains, A/Sw/La Reunion/0164/10 and A/Sw/LaReunion/110348/10, were isolated onto MDCK cell cultures (*5*).

During July–December 2010, 11 farms reported influenza-like clinical signs in pigs, and proof of A(H1N1)pdm09 virus infection was established on 3 farms (farms A–C). In June 2010, fattening pigs on farm A were seronegative for A(H1N1)pdm09 virus. In July, when acute respiratory disease was reported among pigs, 12 of 39 fattening pigs (18–21 weeks old) sampled on farm A were still seronegative for A(H1N1)pdm09 virus; however, rRT-PCR results were positive for A(H1N1)pdm09 virus. Four weeks later, when pigs had recovered from influenza, only 7.7% (3/39) of sampled pigs on farm A had rRT-PCR results positive for A(H1N1)pdm09 virus, and all 39 were seropositive for the virus. High rates of rRT-PCR positivity were also noted for pigs on farms B (17/30 pigs) and C (6/15 pigs). Two A(H1N1)pdm09 strains (A/Sw/LaReunion/0167/10 and A/Sw/La Reunion/110194/10) were isolated from pigs on farms A and B, respectively.

Four influenza virus strains were isolated from pigs, and all induced a cytopathic effect and displayed hemagglutinating activity on chicken erythrocytes; all 4 were confirmed as A(H1N1)pdm09 virus by specific rRT-PCRs. In addition cross-HI assays (*5*) revealed that these strains exhibit antigenic relationships with swine influenza A(H1N1) viruses from classical and avian-like lineages, although they reacted most strongly with A(H1N1)pdm09 virus (Table 1). Genome sequencing of these strains showed high (>98%) nucleotide sequence homology to the corresponding genes of A/California/04/09 and 2009 human strains from Réunion Island, suggesting human-to-swine transmission (H. Pascalis, unpub. data).

In a third step (March, July–August, and October 2011), 3 other sampling campaigns were conducted at the slaughterhouse, including 831 fattening pigs from 104 farms. Nasal swab samples for 7 (8.4%) pigs from 3 (2.9%) farms still had rRT-PCR–positive results. However, serologic analyses revealed that pigs on ≈40% of the farms (distributed throughout the island) were seropositive for A(H1N1)pdm09 virus, indicating continuing circulation of the virus in swine herds (Table 2).

**Table 2:** Seroprevalence rates for influenza A viruses among fattening pigs as determined by ELISA and HI testing, Reunion Island, 2010 and 2011*

Time of sampling campaign (total no. farms; total no. pigs)	Antinucleoprotein ELISA			A(H1N1)pdm09 HI test	
	Herd, %	Pigs, %		Herd†, %	Pigs, %
June 2010 (N = 45; n = 252)	13.33	5.55		8.89	3.57
March 2011 (N = 33; n = 256)	48.48	28.12		42.42	16.41
July–August 2011 (N = 38; n = 316)	47.37	34.49		39.47	27.21
October 2011 (N = 33; n = 259)	57.57	40.93		42.42	22.01

*Pigs were randomly sampled at the slaughterhouse during 4 campaigns. Seroprevalence rates are % farms positive at the herd level among all farms (N) and % pigs positive at the animal level among all animals (n). HI, hemagglutination inhibition. †Within-herd prevalence of infected pigs ranged from 3% to 23%.

## Conclusions

Consistent with findings elsewhere (*8*), our results show that A(H1N1)pdm09 virus has substantially affected swine herds in Réunion Island. Results of our long-term (≈2 years) investigation show that A(H1N1)pdm09 virus has circulated in pigs beyond the 5-week epidemic among humans during the austral winter 2009 (*3*) and has become a novel enzootic pathogen in Réunion Island.

Several facts may account for the heavy human-to-swine transmission of A(H1N1)pdm09 virus. First, the reassortant pandemic virus contains genomic segments originating from swine influenza viruses established in pigs (*1*). Second, pigs are highly susceptible to experimental inoculations with A(H1N1)pdm09 virus and support high intraspecies transmissibility (*9*). Third, the pressure of infection caused by A(H1N1)pdm09 virus among humans in Réunion Island was high but most infections were mild or asymptomatic (*3*); therefore, people pursued their professional activities, acting as silent spreaders of the virus. Last, pigs on Réunion Island had no history of previous passages of swine influenza viruses; thus, the lack of specific immunity to influenza A viruses would have contributed to the high sensitivity of the pigs to infection, as described (*10*,*11*).

Despite serologic proof of large numbers of infected pigs during late 2009–early 2010 in Réunion Island, influenza-like signs were not exhibited and reported until July 2010; this finding was similar to that in New Caledonia (*8*). In July 2010, several herds showed symptomatic changes in infection that could indicate either a change in virulence of the circulating strain or the intervention of co-infecting pathogens or some other environmental factor(s). *Mycoplasma hyopneumoniae* and *Pasteurella multocida* were co-detected on farms with pigs with signs of infection (data not shown). Co-infection with swine influenza virus and these bacteria is known to contribute to severe respiratory disorders among pigs; thus, these bacteria may have enhanced pathogenicity of A(H1N1)pdm09 virus on affected farms (*12*).

Because specific immunity to A(H1N1)pdm09 virus will decline over time when the virus is no longer circulating among humans, persistence of the virus in an animal reservoir raises concerns about the risk for genetic evolution of the virus and retransmission back to humans of variants with potentially increased virulence. As an example, during the 2011 austral winter, only influenza A(H3N2) and B viruses were recorded (*13*). Novel reassortant viruses containing genomic segments from A(H1N1)pdm09 and enzootic swine influenza viruses have been isolated in pigs (*14*). Such a reassortant was responsible for several cases of influenza among humans in 2011 (*15*); these cases were mild, but other, more virulent pathogenic viruses could emerge. Hence continuous surveillance of A(H1N1)pdm09 infection in pigs is recommended.
